# Correction: Epigenetic resetting of human pluripotency (doi:10.1242/dev.146811)

**DOI:** 10.1242/dev.166397

**Published:** 2018-04-18

**Authors:** Ge Guo, Ferdinand von Meyenn, Maria Rostovskaya, James Clarke, Sabine Dietmann, Duncan Baker, Anna Sahakyan, Samuel Myers, Paul Bertone, Wolf Reik, Kathrin Plath, Austin Smith

There were errors in Development (2017) 144, 2748-2763 (doi: 10.1242/dev.146811).

Several images were inadvertently duplicated in Fig. 1F and Fig. S1E. All the original data for these figures were reviewed by the journal and the correct panels are shown below.

**Fig. 1.**
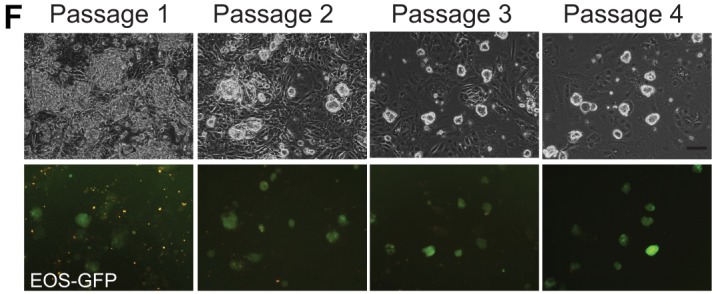
**Resetting human pluripotent stem cells (hPSCs) with HDAC inhibitors.** (F) Images of reset S6EOS cultures over the first four passages. Scale bar: 100 μm.

**Fig. S1.**
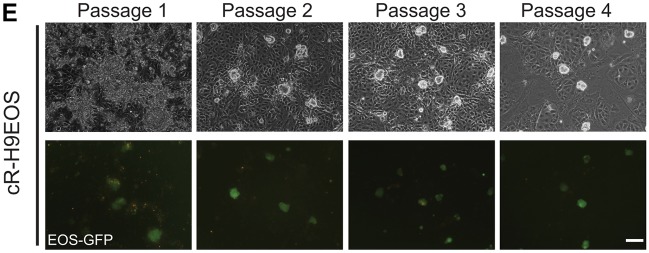
(E) Images of first four passages of reset H9EOS cultures. Scale bar: 100 μm.

This error does not affect the conclusions of the paper. The authors apologise to readers for any confusion caused.

